# Tetherin restricts direct cell-to-cell infection of HIV-1

**DOI:** 10.1186/1742-4690-7-115

**Published:** 2010-12-24

**Authors:** Björn D Kuhl, Richard D Sloan, Daniel A Donahue, Tamara Bar-Magen, Chen Liang, Mark A Wainberg

**Affiliations:** 1 McGill University AIDS Center, Lady Davis Institute, Jewish General Hospital, Montréal, Canada; 2 Department of Experimental Medicine, McGill University, Montréal, Canada; 3 Department of Microbiology and Immunology, McGill University, Montréal, Canada

## Abstract

**Background:**

Tetherin (BST-2/CD317/HM1.24) is an interferon (IFN)-inducible factor of the innate immune system, recently shown to exert antiviral activity against HIV-1 and other enveloped viruses by tethering nascent viral particles to the cell surface, thereby inhibiting viral release. In HIV-1 infection, the viral protein U (Vpu) counteracts this antiviral action by down-modulating tetherin from the cell surface. Viral dissemination between T-cells can occur *via *cell-free transmission or the more efficient direct cell-to-cell route through lipid raft-rich virological synapses, to which tetherin localizes.

**Results:**

We established a flow cytometry-based co-culture assay to distinguish viral transfer from viral transmission and investigated the influence of tetherin on cell-to-cell spread of HIV-1. Sup-T1 cells inducible for tetherin expression were used to examine the impact of effector and target cell tetherin expression on virus transfer and transmission. Using this assay, we showed that tetherin inhibits direct cell-to-cell virus transfer and transmission. Viral Vpu promoted viral transmission from tetherin-expressing cells by down-modulating tetherin from the effector cell surface. Further, we showed that tetherin on the target cell promotes viral transfer and transmission. Viral infectivity in itself was not affected by tetherin.

**Conclusion:**

In addition to inhibiting viral release, tetherin also inhibits direct cell-to-cell spread. Viral protein Vpu counteracts this restriction, outweighing its possible cost of fitness in cell-to-cell transmission. The differential role of tetherin in effector and target cells suggest a role for tetherin in cell-cell contacts and virological synapses.

## Background

Tetherin (BST-2/CD317/HM1.24) is a recently identified component of innate cellular defense against viral infection and is active against HIV-1 and other enveloped viruses [1-5]. Tetherin inhibits viral release from infected cells, tethering nascent viral particles to the cell surface and to each other [3,5,6]. The primary site of action of tetherin is the cellular surface membrane [3,5,7].

In HIV-1 infection, the viral protein Vpu can promote down-modulation of tetherin cell surface expression as well as its subsequent degradation, leading to increased viral release [3,5,8]. Various models have been proposed to link cellular and viral membranes in tetherin-mediated restriction of viral release [3,5,6]. Since tetherin is incorporated into the viral membrane, it may function by directly linking viral and cellular membranes during viral budding through a double anchorage mechanism [6]. It has been suggested that tetherin, in addition to restricting viral release, may also abrogate the infectivity of released HIV-1 particles [9].

Retroviral spread can occur via cell-free and more efficient direct cell-to-cell transmission [10-14] (reviewed in [15,16]). Direct cell-to-cell dissemination between an infected 'effector' cell and an uninfected 'target' cell occurs via intercellular contact zones termed virological synapses that temporarily connect polarized cells [13,17-22]. Virological synapses seem to share structural features with the common immunological synapses that play key roles in cell-mediated immunity [17,19,23-25]. Direct cell-to-cell spread *via *the virological synapse is thought to be a major mode of HIV-1 dissemination in both T-cell lines and in secondary lymphoid tissue [14,20,26-28]. It is possible that cell-to-cell spread may be physically protected from neutralizing antibodies and antiretroviral drugs that target viral entry [14,26,29-33]. Furthermore, it was recently argued that direct cell-to-cell dissemination might be a viral strategy to evade restriction by the innate immune system [34].

Tetherin is an integral membrane protein that combines a conventional transmembrane domain with a glycosyl-phosphatidylinositol (GPI) anchor [35]. At the cell surface, the GPI anchor resides in lipid rafts while the transmembrane domain is thought to localize to the interface of membrane microdomains in ring-like structures [35-38], from where it is down-modulated by Vpu [39]. Lipid raft-rich membrane microdomains were recently shown to be involved in direct cell-to-cell spread of HIV-1 via virological synapses [17,19,40,41]. While cell-free spread of HIV-1 is abrogated by tetherin-mediated restriction of viral release, the accumulation of HIV-1 particles at lipid rafts may alter direct cell-to-cell spread through the virological synapse, as was recently reported for HTLV-1 [42].

Viral infections are also capable of inducing polarization in otherwise non-polarized cells, such as CD4+ T-lymphocytes, in which lipid rafts focus viral entry, assembly and budding [40,41,43,44]. At the virological synapse, virus is recruited to polarized lipid raft domains in transmitting effector cells, while viral receptors necessary for attachment and entry are recruited to the synapse of the target cell in an actin-dependent manner [13,19]. Disturbance of lipid rafts inhibits viral particle production [45,46] and Vpu-mediated viral release [47], as well as the formation of virological synapses [19].

Tetherin has recently been shown to modulate actin cytoskeletal structures in both polarized and non-polarized cells [37]. The structure and localization of tetherin further suggest that it may act as a physical link between cytoskeleton architecture and the plasma membrane in lipid rafts [35-38]. However, little is known about the role of tetherin in virological synapses and the impact of tetherin cell surface expression in effector and target cells on direct cell-to-cell transfer and transmission of HIV-1. Two recent studies reported contradicting data on the role of tetherin in cell-to-cell spread of HIV-1 [48,49]. One study described an inhibiting effect of tetherin on cell-to-cell spread of HIV-1 in absence of Vpu, while also abrogating viral infectivity of transferred virus [49]. Another study reported that tetherin does not restrict HIV-1 cell-to-cell spread, irrespective of Vpu, and also reported an increase of synapse formation with enriched tetherin content at the synapse in the absence of Vpu [48].

Here, we have investigated the impact of cell surface tetherin on HIV-1 cell-to-cell spread using a T-cell line (human T-cell lymphoma cell line Sup-T1), that is inducible for tetherin expression. We found that the presence of tetherin on effector cells diminished HIV-1 cell-to-cell transfer and transmission, and that this activity could be antagonized by Vpu. However, when effector cells lacked tetherin expression, Δ*vpu *virus spread more efficiently than *wt *virus. When expressed on target cells, tetherin promoted viral cell-to-cell transfer and transmission. Tetherin did not exert a direct effect upon the infectiousness of transferred virus.

## Methods

### Cells and viruses

Sup-T1 cells containing the human tetherin gene (tetherin^pos^) as well as negative control cells (tetherin^neg^), *i.e*. cells transduced with an empty vector, have been previously described, and Vpu-dependence of viral release in tetherin^pos ^cells has been confirmed [9]. Cells were maintained in RPMI-1640 supplemented with 10% tetracycline-free bovine serum albumin (BSA), 2 μg/ml puromycin (Sigma), and 1 mg/ml G418 (Sigma). Tetherin expression was induced by adding 0.1 μg/ml doxycycline (Sigma). Cell surface expression of tetherin was assessed by flow cytometry. The viral clone pBR-NL43-IRES-eGFP was obtained from the NIH AIDS Research and Reference Reagent Program. This viral clone expresses green fluorescent protein (GFP) from an internal ribosomal entry site downstream of *nef *[50]. Site-directed mutagenesis, using the QuickChange II XL Site-Directed Mutagenesis Kit (Stratagene), was used to introduce nucleotide changes into the coding region of *vpu*, resulting in two stop codons at amino acid positions 1 and 3 (pBR-NL43-IRES-eGFP Δ*vpu*). Virus was produced in 293T cells using Lipofectamine2000 (Invitrogen) as a transfection reagent. Virus was collected after 48 h, filtered (0.45 μm), and viral capsid/p24 protein (CA p24) content was quantified by VIRONOSTIKA HIV-1 Ag kit (bioMérieux).

### HIV-1 infections

For experiments on cell-to-cell transmission, effector cells (tetherin^pos ^or tetherin^neg^) were infected with 600 ng CA p24/10^6 ^cells by spinoculation (1,500 × g, at 37°C, 2 h), followed by incubation for 1 h at 37°C, after which virus was removed. The spinocultion method was used to synchronize infections. Cells were cultivated for 48 h, at which time the cell population contained 10-12% GFP^pos ^cells as assessed by flow cytometry, thus minimizing superinfection events. To study initial infection kinetics, cells were infected with 350 ng CA p24/10^6 ^cells.

### Western Blot analysis

To verify the absence of Vpu production from the Δ*vpu *viral clone, cells were infected with both *wt *virus and a Δ*vpu *viral clone. Western blots of cellular lysates were probed with antibodies against the viral proteins Vpu (rabbit, NIH AIDS Research and Reference Reagent Program [51]) and CA p24 (mouse, ID Labs Inc.), followed by use of secondary horseradish peroxidase-conjugated secondary antibodies (Sigma).

Expression levels of viral Env and CA p24 in viral particles were assessed from the supernatants of transfected 293T cells (described above). Viral particles were enriched by ultracentrifugation (48,000 × g, 1 h, 4°C). Viral lysates were probed with primary antibodies against Env (rabbit, Abnova) and CA p24 (mouse, ID Lab Inc.) as well as horseradish peroxidase-conjugated secondary antibodies matching the origin of the primary antibody (Sigma). Quantification of viral Env, relative to CA p24, was performed using ImageJ software, following the manufacturer's protocol for ImageJ Gel Analysis documentation.

### Intracellular and extracellular staining

For flow cytometry cells were stained for tetherin on the cell surface and for intracellular CA p24. Staining for cell surface tetherin was performed using a primary rabbit anti-human-tetherin polyclonal antibody (1:3000) (NIH AIDS Research and Reference Reagent Program [52]), followed by a peridinin chlorophyll protein (PerCP)-labeled secondary goat anti-rabbit antibody (1:250) (Santa Cruz Biotechnology). Cells were fixed in 4% paraformaldehyde for 25 min, permeabilized using saponin-containing Wash/Perm solution (BD Bioscience), and stained for intracellular Gag CA p24 using an RD1-labeled mouse anti-CA p24 monoclonal antibody (1:100) (Beckman Coulter). Cell surface staining and intracellular staining were performed at 4°C for 30 minutes. Samples were analyzed by flow cytometry.

For confocal microscopy cells were stained for tetherin and actin; the virus derived GFP signal was amplified by GFP specific staining. Cells were seeded and fixed on coverslips in 4% paraformaldehyde for 25 min and were stained for cell surface tetherin using a primary rabbit anti-human-tetherin antibody (1:3000), followed by addition of anti-rabbit Alex 647-labeled antibody (Invitrogen; 1:400). Cells were then permeabilized using Wash/Perm solution and incubated with Alexa 594-labeled phaloidin (Invitrogen; 1 unit) and anti-GFP Alexa 488-labeled antibody (Invitrogen; 1:400). Cells were scanned using a Zeiss LSM 5 Pascal microscope.

### Analysis of cell-free viral infections, cell-to-cell transfer and transmission by flow cytometry

To assess the impact of tetherin and Vpu on the kinetics of initial viral infection, cells (tetherin^pos ^or tetherin^neg^) were infected with *wt *or Δ*vpu *virus. Expression levels of viral-derived GFP were determined during the initial 48 h of infection.

To investigate the impact of tetherin on cell-to-cell transfer and transmission, effector cells were infected with *wt *or Δ*vpu *virus 48 h prior to setting up co-culture. Target cells were stained with 5 μM 7-amino-4-chloromethylcoumarin (CMAC) (Molecular Probes) at 37°C for 25 min 24 h prior to starting the co-cultivation. Effector and target cells were seeded at a 2:1 ratio to a final concentration of 0.9 × 10^6 ^cells/ml in a final volume of 2 ml in 12-well plates, either in mixed co-culture or separated in transwell chambers with a virus-permeable membrane (3 μm pore size) (NUNC). Virus transfer was assessed by flow cytometry for viral CA p24 in target cells at 6 h after the start of co-culture; virus transmission was evaluated by flow cytometry for virus-derived GFP expression in target cells after 30 h of co-culture. All samples were analyzed using a LSRII instrument (Becton Dickinson), and FACSDiva 6.1 software (Becton Dickinson) or FlowJo 7.5 software (Tree Star).

### Data analysis

Results of at least three independent experiments are expressed as means ± standard error of the mean (SEM). Data were analyzed utilizing GraphPad PRISM 5 software. Differences between two groups were tested for statistical significance using a t-test, while differences between groups of three and more were tested for statistical significance using one-way ANOVA. The p-value obtained from group analyses reflects the overall significance of differences between experimental groups and control groups. Statistical differences between individual groups and their respective control are not stated as exact p-values.

## Results

### Vpu down-modulates induced tetherin cell surface expression in a stably transduced T-cell line

**Figure 1:**
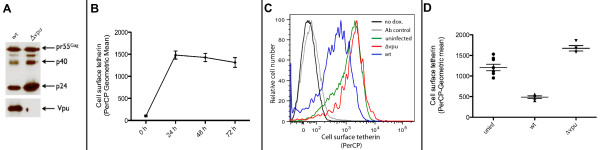
**Vpu down-modulates induced tetherin cell surface expression of tetherin^pos ^Sup-T1 cells**. *A*. Western blot for Vpu (bottom) and CA p24 (top) of *wt- *and Δ*vpu*-infected cells. *B*. Tetherin expression levels upon induction over the course of 72 h. Tetherin cell surface expression was induced by 100 ng/ml doxycycline and was detected by flow cytometry using a PerCP-labeled secondary antibody directed against a primary anti-tetherin antibody. Data points are derived from three independent experiments. *C*. Histogram plot of representative tetherin cell surface expression of non-induced (no dox./black) and induced (100 ng/ml) Sup-T1 cells (green), and induced cells infected with *wt *(blue) and Δ*vpu *(red) BR-NL43-IRES-eGFP viral clones, as well as control cells stained only with PerCP-labeled secondary antibody (Ab control/grey). Cells were gated for infections via GFP expression as a marker for viral gene expression, 48 h post infection. *D*. Geometric means ± standard error of the mean (SEM) of tetherin cell surface expression in uninfected cells and cells infected with *wt *and Δ*vpu *BR-NL43-IRES-eGFP viral clone. Data are derived from three independent experiments; error bars represent SEM.

We first confirmed in our system that Vpu was not expressed from a Δ*vpu *viral clone by Western blot (Figure 1A) and then established the ability of the human T-cell line, Sup-T1, stably transduced with human tetherin (tetherin^pos^), to express tetherin on its cell surface by flow cytometry upon induction by doxycycline [9]. Cell surface expression of tetherin was induced in tetherin^pos ^cells, but not in control Sup-T1 cells (tetherin^neg^) (stably transduced with an empty vector), following addition of doxycycline [9] and established that induced tetherin is stably expressed on the cell surface for at least 72 h (p > 0.4; Figure 1B). Next, we assessed the effect of Vpu on cell surface expression of tetherin in cell populations that were infected with GFP-encoding *wt *or Δ*vpu *BR-NL43-IRES-eGFP viral clone by flow cytometry. Cells were gated into infected and uninfected populations at 48 h post infection (p.i.) based on presence of GFP, as a marker for viral gene expression from BR-NL43-IRES-eGFP. Tetherin surface levels were determined for infected and uninfected cells. In tetherin^pos ^cells infected with *wt *virus, tetherin surface levels were found to be down-regulated by 61% when compared to uninfected cells (p > 0.0001), while tetherin^pos ^cells infected with Δ*vpu *virus showed high levels of surface expression (Figure 1, C and 1D). Modulation of tetherin due to infection was not detected in tetherin^neg ^cells, since levels of cell surface tetherin were below levels of detection at baseline.

### Tetherin localizes to cell-cell contacts

**Figure 2:**
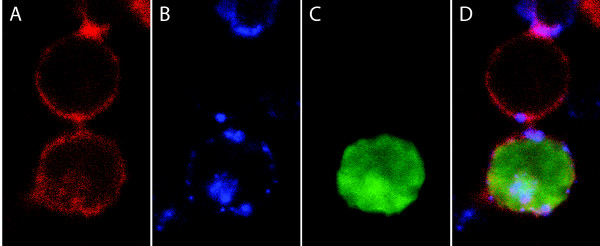
**Tetherin co-localizes with actin at cell-cell contact zones**. Confocal microscopy of co-cultured infected and uninfected tetherin^pos ^cells, stained for actin, cell surface tetherin, and GFP, presented as single stains (*A-C*) and in an overlay image (*D*). Tetherin expression was induced by 100 ng/ml doxycycline and detected using Alexa 647-labeled secondary antibody directed against a primary anti-tetherin antibody (*B*). Actin was stained with Alexa 594 phalloidin (*A*); the virus derived GFP signal was amplified by GFP specific Alexa 488-labeled antibody (*C*).

Tetherin might play a role in direct cell-to-cell spread of HIV-1 in T-cells. We have confirmed that tetherin localizes on both sides of the contact zones between infected and uninfected tetherin^pos ^cells by confocal microscopy/immunofluorescence (Figure 2, bottom and middle), as well as between uninfected cells (Figure 2, middle and top). Further, tetherin co-localizes with actin in the contact zones (Figure 2).

### Equivalence of initial *wt *and Δvpu infection kinetics in tetherin^pos ^and tetherin^neg ^cells

**Figure 3:**
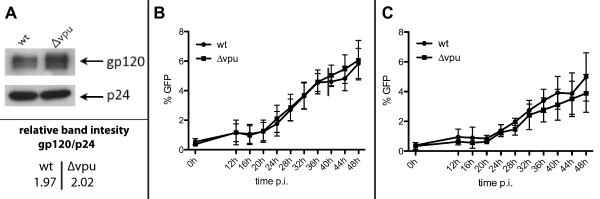
**Neither tetherin nor Vpu affect viral kinetics of initial cell-free infection**. *A*. Shown are representative Western blots of material derived from *wt *and Δ*vpu *viral particles stained for Env and CA p24 (top), as well as Env band intensity values, relative to CA p24, derived by quantification of the representative Western blot using ImageJ software (bottom). Supernatants from 293T transfections were ultracentrifuged and viral extracts analyzed by Western blot, normalized for CA p24. *B & C *Tetherin^pos ^(*B*) and tetherin^neg ^(*C*) Sup-T1 cells were infected with *wt *or Δ*vpu *virus by spinoculation and cells were monitored for virus-derived GFP expression by flow cytometry. Data are derived from three independent experiments; error bars represent SEM.

As the viral genes *vpu *and *env *are present in overlapping fashion in the HIV-1 genome, we investigated whether suppression of Vpu expression might also impact the expression of Env. Western blots from viral extracts confirmed that similar levels of Env were expressed by both the *wt *and Δ*vpu *viral clones, confirmed by similar Env band intensity, quantified relative to CA p24, for *wt *(relative value: 1.97) and Δ*vpu *virus (relative value: 2.02) (Figure 3A). We then performed a flow cytometry-based kinetic analysis of viral infection with both the *wt *and Δ*vpu *viral clones in tetherin^pos ^and tetherin^neg ^cells during an initial 48 h post-infection. Cells were infected with equal amounts of virus, as determined by CA p24, and monitored for viral-derived GFP expression immediately after infection and then at 4 h intervals starting at 12 h post-infection (Figure 3, B and 3C). Statistical analysis of results obtained did not reveal significant differences in regard to the presence/absence of Vpu and tetherin (p > 0.5 for all time points). This further confirms similar levels of Env expression in *wt *and Δ*vpu *viral clones; variations in Env expression might otherwise impact on viral infection.

### Establishment of a cell culture model to assess the effect of tetherin on viral cell-to-cell transfer and transmission

**Figure 4:**
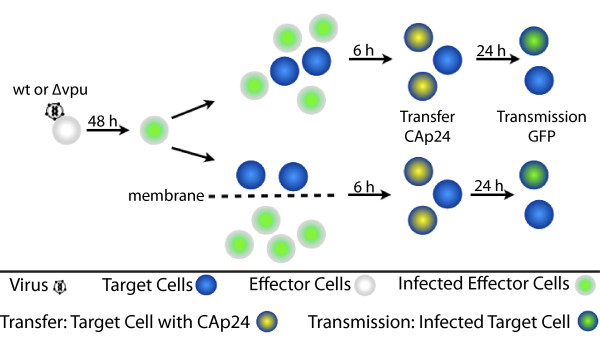
**Co-culture strategy and flow cytometry analysis**. Effector cells (grey) were infected with *wt *or Δ*vpu *virus, cultured for 48 h, washed and then co-cultured with uninfected target cells, which were stained with CMAC (blue) either together or separated by a virus permeable membrane (3 μm pore size). Viral transfer was assessed by flow cytometry analysis of viral CA p24 protein (yellow) in target cells 6 h after the initiation of co-culture. Viral transmission was assessed 24 h later by flow cytometry analysis for GFP expression (green) in target cells.

We then set out to establish an assay that would assess the impact of effector and target cell tetherin cell surface expression on direct viral cell-to-cell spread, discriminating between viral transfer and viral transmission. Tetherin^pos ^and tetherin^neg ^cells were infected with equal amounts of *wt *or Δ*vpu *virus (based on ng CA p24) and cultured for 48 h, resulting in 10-12% infected cells, as determined by measurement of GFP expression by flow cytometry. Cells were washed, then co-cultured with uninfected cells at a 2:1 (infected:uninfected) cell ratio, which has been reported to increase viral spread [26]. Uninfected tetherin^pos ^and tetherin^neg ^cells were stained with CMAC, a dye allowing the tracking of uninfected cells over multiple cell divisions, from 24 h prior to the start of co-culture. Cells were then co-cultured together to allow cell-cell contact or separated by a membrane (3 μm pore size) in a transwell system (Figure 4) that can exclude cells while permitting free viral diffusion (data not shown) [26]. Samples were collected at 6 h and 30 h after the initiation of co-culture to assess viral transfer and transmission, respectively. After 6 h, viral CA p24 was detected in target cells by flow cytometry, indicating viral transfer, while expression of virus-derived GFP was not observed. After 30 h, GFP was detected, indicating that viral transmission had occurred.

Cells were stained for intracellular CA p24 and cell surface tetherin. Flow cytometry analysis was performed for CMAC (to identify previously uninfected target cells), viral derived GFP, viral CA p24 and cell surface tetherin. Cells were gated for live and single cells, followed by gating for a CMAC positive target cell population. The detection of viral CA p24 in this population after 6 h allows identification and quantification of viral transfer, while GFP expression in this population at 30 h is a measurement of virus transmission. While other groups have detected viral transmission based on intracellular CA p24 levels, we assessed viral transmission in terms the detection of virus-derived eGFP. Since CA p24 expression in infected cells can *per se *not be distinguished from CA p24 derived from viral transfer, the CA p24-based transmission detection method relies on controls, wherein synthesis of CA p24 is diminished by antiviral drugs [26]. Our data show that our system, which does not rely on drug controls, is at least as sensitive in regard to detection of viral transmission (Supplementary Figure 1). All experiments described below examine viral transfer and transmission in the direct co-culture, in which cell-cell (effector-target) contact can occur. Examination of target cells in the transwell co-cultures served as a control to show that cell-free viral dissemination transfer was inefficient in the absence of direct cell-cell contact [14]. While we present and discuss normalized data below, original readouts are presented in Supplementary Figure 2.

### Tetherin expression on effector cells diminishes both viral transfer and transmission and is antagonized by Vpu

**Figure 5:**
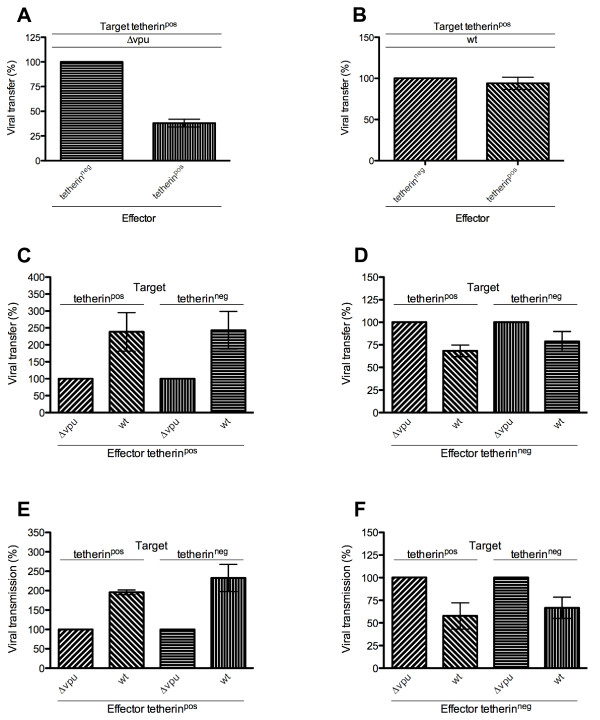
**Tetherin cell surface expression on effector cells inhibits viral cell-to-cell transfer and transmission and is counteracted by Vpu**. Tetherin^pos ^or tetherin^neg ^Sup-T1 effector cells, infected with *wt *or Δ*vpu *virus, were co-cultured with tetherin^pos ^or tetherin^neg ^Sup-T1 target cells. Target cells were assessed for viral transfer and viral transmission by flow cytometry analysis for CA p24 and GFP expression, respectively. Data are derived from three independent experiments; error bars represent SEM. *A & B*. Viral transfer (CA p24) of Δ*vpu *(*A*) and *wt *(*B*) virus from tetherin^pos ^and tetherin^neg ^effector cells. Data are normalized to the number of infected tetherin^neg ^effector cells. *C & D*. Viral transfer (CA p24) of *wt *and Δ*vpu *virus from tetherin^pos ^(*C*) and tetherin^neg ^(*D*) effector cells. Data are normalized for Δ*vpu *infections in each case. *E & F*. Viral transmission (GFP) of *wt *and Δ*vpu *virus from tetherin^pos ^(*E*) and tetherin^neg ^(*F*) effector cells. Data are normalized for Δ*vpu *infections in each case.

Tetherin expression at the surface of effector cells restricted transfer of Δ*vpu *virus to tetherin^pos ^target cells by 61% as assessed by intracellular CA p24 (Figure 5A) (p = 0.0259). In contrast, transfer of *wt *virus was not significantly affected by tetherin expression on the cell surface of effector cells (Figure 5B) (p > 0.5). Therefore, tetherin restricts viral transfer in direct cell-to-cell dissemination; Vpu antagonizes this tetherin-mediated restriction.

We next decided to investigate the impact of tetherin on viral transfer in greater detail by monitoring viral transfer from tetherin^pos ^cells to either tetherin^pos ^or tetherin^neg ^target cells in direct co-culture experiments. The results show that transfer was increased by 140% when Vpu was present (*wt *virus) compared to when Vpu was absent (Δ*vpu*) (p = 0.0235) (Figure 5C). Conversely, Vpu negatively affected viral transfer from tetherin^neg ^to either tetherin^pos ^or tetherin^neg ^cells. Viral transfer in the presence of Vpu (*wt *virus) reached only 70-80% of the levels of transfer that occurred in the absence of Vpu (Δ*vpu*) (p = 0.0235) (Figure 5D). No GFP^pos ^cells were detected in the target cell population, indicating that viral transfer but not transmission had occurred. As flow cytometry detection was performed on non-aggregated single cells, possible fusion events between infected effector cells and target cells might not have been assessed.

No CA p24 was detected after 6 h in any of the potential target cell populations in transwell co-culture experiments, where direct cell-cell contact had been blocked, confirming the relative inefficiency of cell-free viral transmission. An absence of CMAC^pos ^cells in the effector cell population confirmed the integrity of the transwell membrane.

As opposed to viral transfer, transmission and actual infection of new cells must lead to expression of viral genes. To examine the effect of tetherin on viral transmission in direct cell-to-cell spread, cells were co-cultured for 30 h and virus-derived GFP expression in the target cell population was determined. When effector cells expressed tetherin, expression of GFP from *wt *virus in target cells was 200-230% of levels attained with Δ*vpu *virus (p = 0.0235) (Figure 5E). In contrast, when effector cells failed to express surface tetherin, transmission of *wt *virus was apparently reduced to 55-66% of levels attained with Δ*vpu *virus (p = 0.0235) (Figure 5F). No GFP was detected in target cell populations in any transwell culture after 30 h, confirming the relative inefficiency of cell-free viral transmission.

### Tetherin expression on target cells promotes viral transfer and viral transmission

**Figure 6:**
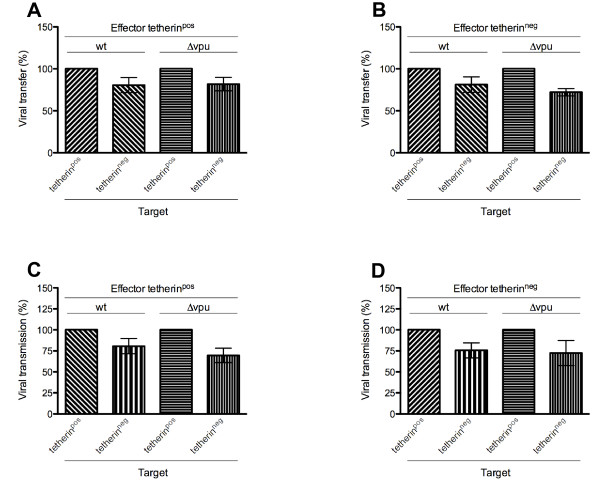
**Tetherin cell surface expression on target cells promotes viral cell-to-cell transfer and transmission**. Tetherin^pos ^or tetherin^neg ^Sup-T1 effector cells, infected with *wt *or Δ*vpu *virus, were co-cultured with tetherin^pos ^or tetherin^neg ^Sup-T1 target cells. Target cells were assessed for viral transfer and viral transmission by flow cytometry analysis for CA p24 and GFP expression, respectively. Data are normalized for tetherin^pos ^target cells in each case. Data are derived from three independent experiments; error bars represent SEM. (*A & B)*. Viral transfer (CA p24) of *wt *and Δ*vpu *virus from tetherin^pos ^(*A*) and tetherin^neg ^(*B*) effector cells. (*C & D)*. Viral transmission (GFP) of *wt *and Δ*vpu *virus from tetherin^pos ^(*C*) and tetherin^neg ^(*D*) effector cells.

We next asked whether tetherin expression on target cells impacts viral cell-to-cell transfer. We found that tetherin expression on target cells significantly increased transfer of both *wt *and Δ*vpu *virus, irrespective of the presence or absence of tetherin on effector cells. When the latter expressed tetherin, transfer of both *wt *and Δ*vpu *virus to tetherin^neg ^target cells was 20% lower than to tetherin^pos ^cells (p = 0.0235) (Figure 6A). Similarly, when effector cells lacked tetherin surface expression, transfer of both *wt *and Δ*vpu *virus to tetherin^neg ^target cells was reduced by 20% and 27%, respectively, compared to transfer to tetherin^pos ^target cells (p = 0.0223) (Figure 6B).

Further to this, we observed 21% and 30% decreases in transmission of *wt *and Δ*vpu *virus from tetherin^pos ^effector cells to tetherin^neg ^target cells, respectively, compared to transmission of these viruses to tetherin^pos ^cells (p = 0.02) (Figure 6C). Similar decreases were obtained for transmission of *wt *(24%) and Δ*vpu *(27%) virus from tetherin^neg ^effector cells (p = 0.0235) (Figure 6D).

### Tetherin does not impact the infectiousness of transferred HIV-1

**Figure 7:**
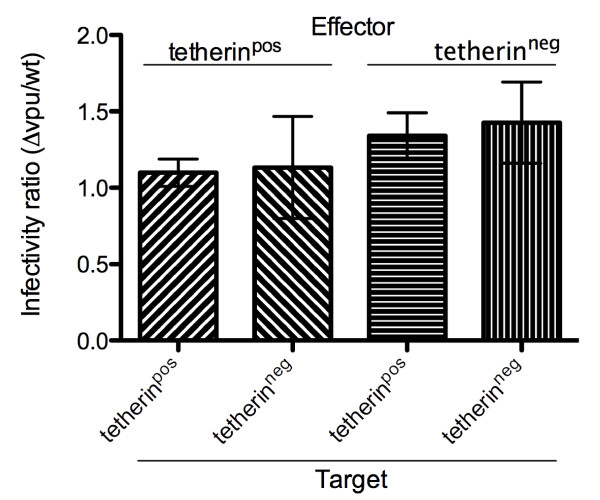
**Tetherin does not affect infectiousness of transferred virus**. Tetherin^pos ^or tetherin^neg ^effector cells, infected with *wt *or Δ*vpu *virus, were co-cultured with tetherin^pos ^or tetherin^neg ^target cells. Ratios of transmission *vs *transfer were calculated; ratios of Δ*vpu *and *wt *infections were compared to determine the impact of tetherin and Vpu on viral infectivity. A ratio of one indicates similar viability of the Δ*vpu *and the *wt *virus. Ratios <1 represent an advantage for Vpu (*wt *virus), while a ratio >1 indicates a fitness cost of Vpu (Δ*vpu *virus) in viral cell-to-cell spread. Data are derived from three independent experiments; error bars represent SEM.

The above result document a differential effect of Vpu on viral transfer and transmission, depending on tetherin surface expression on effector cells, and a Vpu-independent effect of tetherin surface expression on target cells. We next asked whether these factors might impact on viral infectivity in cell-to-cell transmission by calculating ratios of viral transmission and transfer. By deriving transmission *vs*. transfer ratios between *wt *and Δ*vpu *virus, we assessed the effect of Vpu (Figure 7). Significant differences were not observed (p > 0.05), suggesting that cell surface tetherin expression did not affect viral infectiousness in regard to direct cell-to-cell dissemination. However, when effector cells lacked tetherin, the ratio was increased by ~25%, irrespective of the presence or absence of tetherin on target cells. This suggests that there is a fitness cost associated with Vpu in the context of cell-cell spread when effector cells do not express tetherin.

## Discussion

Tetherin is an IFN-α-inducible antiviral factor that functions in innate immunity by linking nascent virus to the cellular surface and inhibiting virus release [3,5]. The Vpu-mediated countermeasure is localized to lipid rafts at the cell surface [3,5,6,35,38,39]. Lipid rafts are sites of polarized cell-cell contact in immunological and virological synapses [13,17,18,20,21,53].

Here, we have investigated how cell surface expression of tetherin on effector and target cells can modulate cell-to-cell transfer and transmission of HIV-1. To this end we used a T-cell line, Sup-T1, inducible for human tetherin expression [9]. In contrast to cell lines or primary cells, wherein tetherin expression can be achieved as part of the cellular antiviral state *via *the multifaceted IFN-response, use of the doxycycline-inducible cell-line allowed us to exclusively investigate the contribution of tetherin on cell-to-cell spread.

In our system, tetherin surface levels in these HIV-1 infected tetherin^pos ^cells were down-regulated in a Vpu-dependent manner (Figure 1), confirming the role of Vpu in counteracting tetherin, for which the underlying mechanisms are not yet fully understood [5,7,9,36,54]. However, infection of these cells with Δ*vpu *virus caused tetherin cell surface levels to be up-regulated when compared to non-infected cells. This observation is not due non-specific staining, as ruled out by internal controls for the primary and secondary antibody (Figure 3C, comparing uninduced (black) and control staining (grey)). We speculate that this effect is most likely due to a tethering of viral particles to the cell surface and subsequent greater accessibility of tetherin to antibody binding, though this hypothesis requires investigation. Another potential explanation could be the cell surface increase in infected cells due to swelling in infected cells.

In the viral genome, the Vpu and Env proteins are encoded in overlapping fashion. As we introduced premature stop codons into the coding region of *vpu *by site-directed mutagenesis to generate a Δ*vpu *viral clone, we assessed and confirmed the similarity of viral Env levels in *wt *and Δ*vpu *viral particles (Figure 3A). Therefore, differences in cell-to-cell spread in regard to Vpu-mediated tetherin downmodulation are independent of potential variation of Env expression. Further, we also confirmed similarity of cell-free viral infection kinetics in tetherin^pos ^and tetherin^neg ^cells (Figure 3, B and 3C)

Confocal microscopy revealed cell surface tetherin focused on cell-cell contact zones between infected and uninfected cells, as well as between uninfected cells, where it co-localized with actin (Figure 2). Therefore, tetherin is adequately positioned to modulate cell-to-cell spread of HIV-1. We established a flow cytometry assay to assess the impact of tetherin cell surface expression on viral cell-to-cell spread (Figure 4). In contrast to other similar assays [26], ours has the advantage of distinguishing between viral transfer and infection in the same cell population. This was accomplished by detection of intracellular viral CA p24 protein as a marker of viral transfer *vs *the expression of virus-derived GFP as a marker of infection. Employing an eGFP-expressing reporter virus is crucial to this assay in order to distinguish transfer from transmission, since transferred CA p24 cannot be easily distinguished from newly synthesized CA p24 by staining and flow cytometry. Rather, this distinction relies on control experiments that employed inhibitors of reverse transcriptase to abrogate new synthesis of CA p24 in infected cells [26]; however, it is not known to what extent cell-to-cell spread may be impervious to inhibitors of reverse transcription, neutralizing antibodies, and inhibitors of viral entry [14,31,33,55]. In our system, the presence of Efavirenz (500 nM) only partially reduced infection of target cells (Supplementary Figure 1). Change in CA p24 mean fluorescence intensity might not directly reflect productive infection (Supplementary Figure 1). In our studies we used a reporter virus, wherein eGFP is encoded by means IRES adjacent to *nef*, and is therefore expressed early in viral infection [56-58]. Therefore, in our system, the absence of GFP in target cells at the transfer time point (6 h) is a valid control; CA p24 detected in target cells at this time is exclusively derived from transfer events. This set up also enabled us to calculate an infectivity ratio, derived from the percentage of cells expressing viral genes after 30 h *versus *the percentage of cells showing evidence of viral transfer after 6 h. Furthermore, a comparison of such ratios using either *wt *or Δ*vpu *viruses allowed us to investigate the interrelationship between Vpu and tetherin in regard to viral cell-to-cell spread.

Using this system, we have shown that induction of tetherin at the effector cell surface significantly diminishes cell-to-cell transfer of Δ*vpu *virus (Figure 5A). Viral Vpu antagonizes this restriction; transfer of *wt *virus was not significantly affected by tetherin (Figure 5B). Therefore, tetherin-mediated accumulation of HIV-1 particles at the cell surface does not increase viral cell-to-cell spread of HIV-1, as occurs with HTLV-1 [42].

We observed 2-fold increases in both viral transfer and transmission of *wt *virus compared to Δ*vpu *virus from tetherin^pos ^effector cells (Figure 5, C and 5E). Recent studies on the effect of Vpu on viral release have shown increases of 2-70 fold as assessed by extracellular CA p24 [3,5,8,9,34]. Direct cell-to-cell spread of HIV may be 100-18,000 times more efficient than cell-free spread [10,14,59].

Our data show that direct viral transfer and transmission were affected by tetherin to similar extents, indicting that the reduction in viral transmission is directly related to the reduction in viral transfer. However, we found that Δ*vpu *virus spread more efficiently from tetherin^neg ^cells than *wt *virus (Figure 5), a result consistent with reports by others of increased transfer of Δ*vpu *virus [26,28].

When assessing the infectivity of the transferred virus in cell-to-cell dissemination, we did not observe an overall statistically significant effect of tetherin that was expressed on the surface of effector cells or of its Vpu-mediated down-modulation. However, we report the apparently increased infectiousness of Δ*vpu *virus when effector cells lacked tetherin expression (Figure 7). This observation, together with the more efficient dissemination of Δvpu virus than *wt *virus from tetherin^neg ^cells (Figure 5), suggests the faster replication of Δ*vpu *virus in this setting (cell-to-cell spread) but not during the initial (cell-free) phase of infection, which proved not to be affected by Vpu, irrespective of the presence/absence of tetherin at the cell surface (Figure 3, B and 3C). This further suggests that Vpu imposes a fitness cost in terms of cell-to-cell viral dissemination when tetherin is absent. This effect seems to be related only to direct cell-to-cell viral spread, but not to cell-free spread, as initial cell-free infections were not affected (Figure 3, B and 3C). This is further supported by the selection of a Δ*vpu *mutant in a co-culture study that used the Jurkat T-cell line [28].

Surprisingly, we found that tetherin expression on target cells led to higher levels of viral transfer and transmission. This effect was independent of tetherin cell surface expression in effector cells (Figure 6). Even though the observed effect was modest, it was found to be statistically significant. As this effect would occur in the setting of highly effective direct cell-to-cell spread, the ultimate impact of even a modest increase in transmission might be large and indicates a likely role for tetherin on target cells in the virological synapse. In cellular membranes, tetherin resides in lipid rafts [35,38]; its expression *via *IFN-α is stimulated as a response to viral infections [5,60]. Tetherin can also modulate the actin cytoskeleton of polarized and non-polarized cells and is thought to link actin architectures and cellular membranes [35,37,38]. It is therefore possible that cell surface tetherin expression modulates the virological synapse and subsequently influences cell-to-cell transfer and transmission, but not cell free infection, which is not dependent on synapse formation. Such modulation could occur through stabilization of synapse structure on both effector and target cell and/or actin-dependent recruitment of viral receptors that are necessary for entry on the target cell [13,61]. This is in agreement with the observed co-localization of tetherin and actin in the contact zone of effector and target cells (Figure 2). Recently, it was proposed that HIV-1 can spread between T-cells *via *actin-containing membrane extensions in a receptor-dependent manner [30,62]. HIV-1 may hijack the IFN-α-mediated innate immune response in order to increase the efficiency of viral spread. These ideas are speculative, based on an understanding of the current literature.

During the preparation of this manuscript, another study reported that tetherin expression restricts cell-to-cell transmission of HIV-1 and that such restriction is antagonized by Vpu [49]. In addition, a different study reported that cell-to-cell HIV spread is not affected by tetherin [48]. Our experiments enabled us to assess viral transfer and transmission in the same cell population, by using a stably transduced cell line that is inducible for tetherin expression for both effector and target cells. Thus, we were able to investigate tetherin-mediated effects independent of differences in cell lines and independent of the complex cellular response to IFN. Although an IFN approach is probably more physiologically relevant, our system has limited the variables to tetherin and Vpu only and shows similar tetherin-mediated effects on transfer and transmission. Although generally in agreement in regard to tetherin-mediated restriction of cell-to-cell viral spread, one study reported a discordance between transfer and transmission, suggesting an impact of tetherin on viral infectivity [49]. While we report results showing that tetherin might reduce the infectivity of the Δ*vpu *virus to levels equivalent to those of wt virus (Figure 7), a different study reported reduced infectivity of a Δ*vpu *virus compared to *wt *and attributed this to tetherin [49]. Others showed an impact of tetherin on the formation of virological synapses [48,49], but did not specifically test for an effect of tetherin in target cells in regards to cell-to-cell spread, as has been performed here. Specifically, one study reported an increase in synapse formation in the presence of tetherin [48], which is supportive of our finding that tetherin promotes cell-to-cell spread when expressed on target cells. This result also supports our hypothesis that tetherin plays a role in synapses and that Vpu might represent a modulator of synapse involvement *via *tetherin. Differences in regard to restriction of cell-to-cell spread of HIV-1 by tetherin [48,49] might be due to differential methodology and analysis (flow cytometry detection of CA p24 and virus-derived eGFP expression compared to flow cytometry detection of changes in CA p24 levels, scanning electron microscopy, and qPCR of RT products). Importantly, there were also differences between these studies in the source of tetherin used as well as tetherin expression levels, and tetherin modulation (stably transduced cell line, inducible for tetherin expression, compared to primary cells, as well as various cell lines with different expression levels, transfections, and IFN stimulation paired with anti-tetherin shRNA). These factors might fundamentally impact on the outcome of the studies, and might also explain the tetherin-mediated effect on infectivity of released virus as recently reported by others [63].

Recent findings that HIV-2 Env and SIV Nef exhibit Vpu-like function in down-modulating tetherin from the cell surface (without down-modulating CD4, in the case of HIV-2) underline the importance of countering tetherin-mediated restriction [64,65]. Some have argued that tetherin activity may be important in restricting cross-species transmission of HIV and its diversification [66]. Although it is accepted that tetherin restricts the cell-free dissemination of HIV-1, it will be of importance to further address whether tetherin can affect other aspects of cell-to-cell spread, which can occur with high efficiency. Our data provide support for a role of tetherin in restricting the cell-to-cell spread of HIV-1 [49].

## Conclusions

We have shown that tetherin, in addition to limiting cell-free viral spread, also restricts direct cell-to-cell spread of HIV-1. Virus transfer and transmission were both affected by tetherin and restriction, in each case, would be overcome by the viral protein Vpu, which down-modulates tetherin from the cell surface. The observation that Vpu is necessary for cell-to-cell spread from tetherin-expressing cells, but is disadvantageous in tetherin-free settings, suggests that Vpu presents a fitness cost to the virus in regard to cell-to-cell spread, that is outweighed by its ability to antagonize tetherin. The differential role of tetherin in effector and target cells suggests a role for tetherin in cell-cell contacts and virological synapses. Targeting Vpu and promoting tetherin-mediated restriction should be advanced as an antiviral strategy.

## Competing interests

The authors declare that they have no competing interests.

## Authors' contributions

BDK performed all of the work in this manuscript in partial fulfillment of the requirements of a Ph.D. degree, Faculty of Graduate Studies and Research, McGill University, Montreal, Canada. BDK also composed the first draft of this manuscript. RDS co-supervised the work described and reviewed the manuscript. DAD contributed constructs and ideas for the work. T B-M co-supervised this work and reviewed the manuscript. CL contributed constructs and reviewed the manuscript. MAW was the primary supervisor of this work and reviewed and corrected the manuscript.

## Supplementary Material

Additional file 1 **Supplementary Figure 1. Evaluation of sensitivity of detection of transmission**. Virus transmission in untreated target cells and in cells treated with Efavirenz (EFV; 500 nM) was based on the detection of intracellular CA p24 or virus derived eGFP at 30 h of co-culture with infected effector cells. *A & B*. Percentages of cells gated positive for CA p24 (*A*) or eGFP (*B*) in the absence or presence of EFV. Differences between the EFV-treated and untreated cells were statistically significant (*A*: p = 0.0005; *B*: p = 0.0001; Student's t-test). Data show three independent experiments; error bars represent SEM. *C*. Detection of eGFP is more sensitive than detection of CA p24. Shown is a summary of means ± SEM, differences of means ± SEM, and differences normalized to the no-drug control (% change) from cells gated positive for CA p24 and eGFP. *D-G*. Changes in mean fluorescence intensity might not reflect infection. Shown are target cells gated positive for CA p24 (red) and eGFP (blue), in regard CAp24 fluorescence (% cells of target cell population) (*D & F*) and mean fluorescence intensity (geometric means) (*E & G*). Cells were cultured in the absence (*D & E*) or the presence of EFV (500 nM; *F & G*). While eGFP positive target cells clearly show high CA p24 levels in the absence of drug (*D & E*), eGFP positive cells are also present in the drug-treated target cell population (*F*); geometric mean differences between eGFP positive and CA p24 positive, drug-treated, target cells are less pronounced (*G*) and might lead to an underestimate of transmission events. Therefore, the detection of transmission events might be less sensitive than observed through direct detection of virus-derived eGFP. Click here for file

Additional file 2 **Supplementary Figure 2. Original data from three independent experiments**. Shown are tabular data of transfer (top), transmission (middle) and infectivity ratios (bottom) for three experiments (Experiments 1-3); numbers represent % of target cell population. Virus transfer was detected by intracellular staining for CA p24 in target cells at 6 h of co-culture; transmission was assessed *via *detection of virus-derived eGFP expression in target cells at 30 h of co-culture. Click here for file
