# Supplementary figures and images for: Tetherin restricts direct cell-to-cell infection of HIV-1
Source: Retrovirology. 2010 Dec 24;7:115. doi: 10.1186/1742-4690-7-115 (PMC3017029; doi:10.1186/1742-4690-7-115)

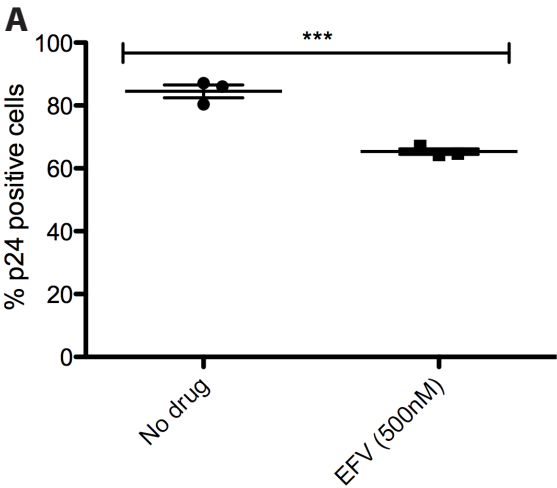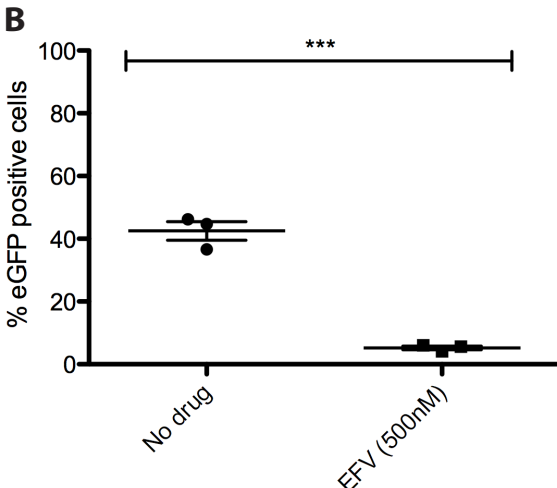

**C**

| Treatment \ Detection | No drug       | EFV (0.5μM)    | Δ of means    | % of change |
|-----------------------|---------------|----------------|---------------|-------------|
| p24 (Geometric mean)  | 84.50 ± 2.074 | 65.37 ± 0.8743 | 19.13 ± 2.251 | 32 %        |
| eGFP (Geometric mean) | 42.50 ± 2.982 | 5.233 ± 0.5783 | 37.27 ± 3.037 | 87 %        |

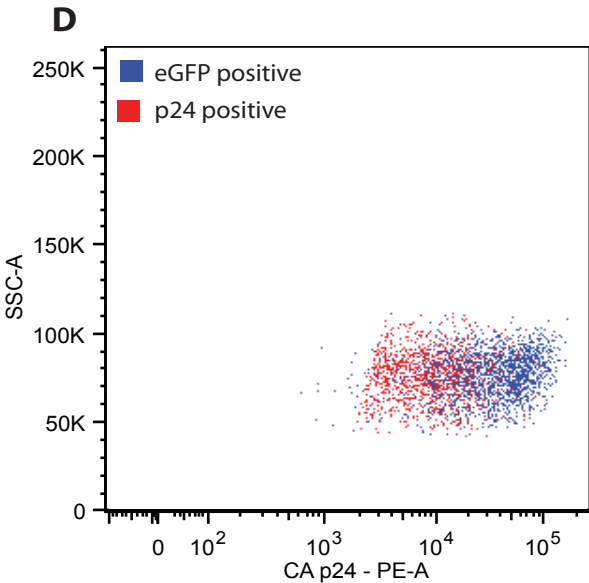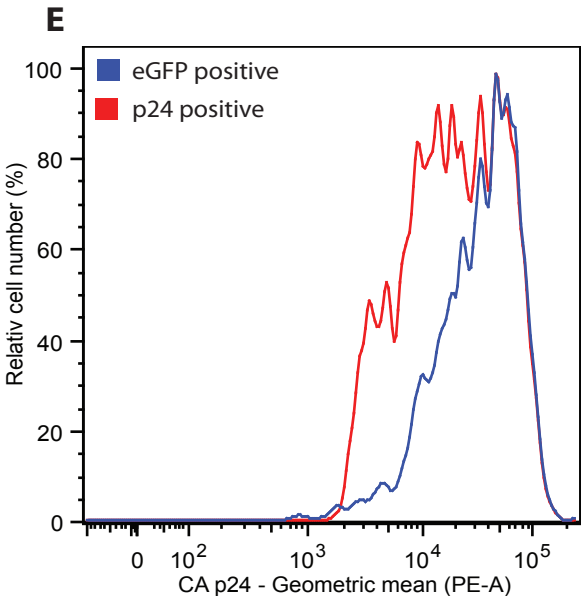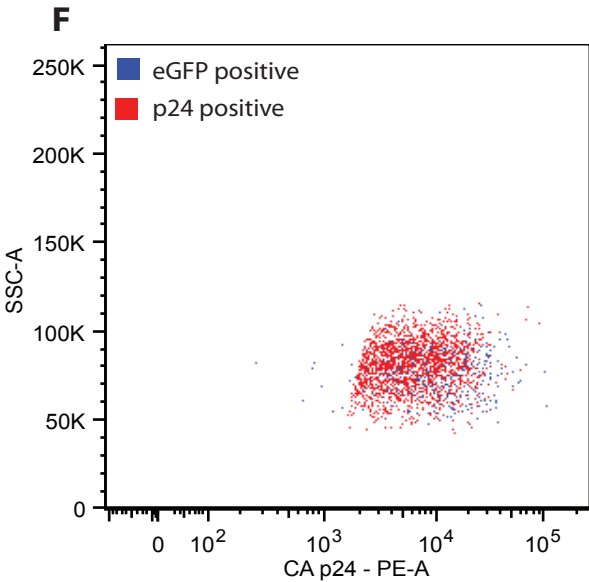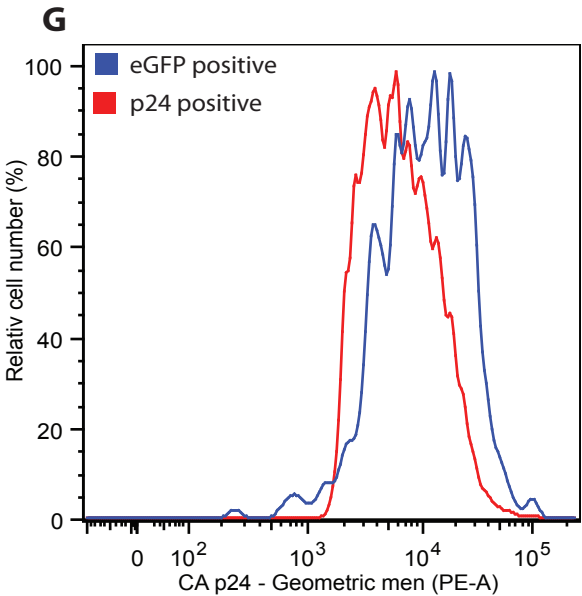

Supplement: Additional file 1 — Supplementary Figure 1. Evaluation of sensitivity of detection of transmission. Virus transmission in untreated target cells and in cells treated with Efavirenz (EFV; 500 nM) was based on the detection of intracellular CA p24 or virus derived eGFP at 30 h of co-culture with infected effector cells. A & B. Percentages of cells gated positive for CA p24 (A) or eGFP (B) in the absence or presence of EFV. Differences between the EFV-treated and untreated cells were statistically significant (A: p = 0.0005; B: p = 0.0001; Student's t-test). Data show three independent experiments; error bars represent SEM. C. Detection of eGFP is more sensitive than detection of CA p24. Shown is a summary of means ± SEM, differences of means ± SEM, and differences normalized to the no-drug control (% change) from cells gated positive for CA p24 and eGFP. D-G. Changes in mean fluorescence intensity might not reflect infection. Shown are target cells gated positive for CA p24 (red) and eGFP (blue), in regard CAp24 fluorescence (% cells of target cell population) (D & F) and mean fluorescence intensity (geometric means) (E & G). Cells were cultured in the absence (D & E) or the presence of EFV (500 nM; F & G). While eGFP positive target cells clearly show high CA p24 levels in the absence of drug (D & E), eGFP positive cells are also present in the drug-treated target cell population (F); geometric mean differences between eGFP positive and CA p24 positive, drug-treated, target cells are less pronounced (G) and might lead to an underestimate of transmission events. Therefore, the detection of transmission events might be less sensitive than observed through direct detection of virus-derived eGFP. [file 1742-4690-7-115-S1.PDF]
