# Supplementary material for: Tetherin restricts direct cell-to-cell infection of HIV-1
Source: Retrovirology. 2010 Dec 24;7:115. doi: 10.1186/1742-4690-7-115 (PMC3017029; doi:10.1186/1742-4690-7-115)
Supplement: Additional file 2 — Supplementary Figure 2. Original data from three independent experiments. Shown are tabular data of transfer (top), transmission (middle) and infectivity ratios (bottom) for three experiments (Experiments 1-3); numbers represent % of target cell population. Virus transfer was detected by intracellular staining for CA p24 in target cells at 6 h of co-culture; transmission was assessed via detection of virus-derived eGFP expression in target cells at 30 h of co-culture. [file 1742-4690-7-115-S2.PDF]

Experiment 1

Virus transfer. Detection of intracellular Gag in target cells at 6h.

Cell / Virus	Effector	$tetherin^{pos}$		$tetherin^{neg}$	
	Target	$tetherin^{pos}$	$tetherin^{neg}$	$tetherin^{pos}$	$tetherin^{neg}$
wt		4.3	3.2	2.7	1.9
Δvpu		2.7	2.1	4.8	4.1

Virus transmission. Detection of virus-derived eGFP in target cells at 30h.

Cell / Virus	Effector	$tetherin^{pos}$		$tetherin^{neg}$	
	Target	$tetherin^{pos}$	$tetherin^{neg}$	$tetherin^{pos}$	$tetherin^{neg}$
wt		6.8	6.7	10.9	10.2
Δvpu		3.6	2.4	17.1	13

Viral infectivity. Ratio of viral transfer and transmission data.

Cell / Virus	Effector	$tetherin^{pos}$		$tetherin^{neg}$	
	Target	$tetherin^{pos}$	$tetherin^{neg}$	$tetherin^{pos}$	$tetherin^{neg}$
wt		0.63	0.47	0.25	0.19
Δvpu		0.75	0.85	0.28	0.31
**Δvpu/wt**		**1.19**	**1.8**	**1.12**	**1.63**

Experiment 2

Virus transfer. Detection of intracellular Gag in target cells at 6h.

Virus \ Cell	Effector	tetherin^{pos}		tetherin^{neg}	
	Target	tetherin^{pos}	tetherin^{neg}	tetherin^{pos}	tetherin^{neg}
wt		11	7.9	3.8	2.4
Δvpu		6.3	5.9	6.3	4

Virus transmission. Detection of virus-derived eGFP in target cells at 30h.

Virus \ Cell	Effector	tetherin^{pos}		tetherin^{neg}	
	Target	tetherin^{pos}	tetherin^{neg}	tetherin^{pos}	tetherin^{neg}
wt		9.3	6.9	1.8	1.2
Δvpu		4.9	4.2	4	1.8

Viral infectivity. Ratio of viral transfer and transmission data.

Virus \ Cell	Effector	tetherin^{pos}		tetherin^{neg}	
	Target	tetherin^{pos}	tetherin^{neg}	tetherin^{pos}	tetherin^{neg}
wt		0.85	0.87	0.47	0.5
Δvpu		0.78	0.71	0.63	0.45
**Δvpu/wt**		**0.92**	**0.82**	**1.3**	**0.9**

Experiment 3

Virus transfer. Detection of intracellular Gag in target cells at 6h.

Virus \ Cell: Effector	tetherin^{pos}		tetherin^{neg}	
Target	tetherin^{pos}	tetherin^{neg}	tetherin^{pos}	tetherin^{neg}
wt	20.6	15.1	19.4	14.4
Δvpu	8.4	7.4	9.7	7.4

Virus transmission. Detection of virus-derived eGFP in target cells at 30h.

Virus \ Cell: Effector	tetherin^{pos}		tetherin^{neg}	
Target	tetherin^{pos}	tetherin^{neg}	tetherin^{pos}	tetherin^{neg}
wt	8.1	5.6	5.2	4.1
Δvpu	3.9	2.2	4.3	3.6

Viral infectivity. Ratio of viral transfer and transmission data.

Virus \ Cell: Effector	tetherin^{pos}		tetherin^{neg}	
Target	tetherin^{pos}	tetherin^{neg}	tetherin^{pos}	tetherin^{neg}
wt	0.39	0.37	0.27	0.28
Δvpu	0.46	0.29	0.44	0.49
**Δvpu/wt**	**1.18**	**0.78**	**1.62**	**1.75**
